# Individual clown anemonefish shrink to survive heat stress and social conflict

**DOI:** 10.1126/sciadv.adt7079

**Published:** 2025-05-21

**Authors:** Melissa A. Versteeg, Chancey MacDonald, Morgan F. Bennett-Smith, Peter M. Buston, Theresa Rueger

**Affiliations:** ^1^ Dove Marine Laboratory, School of Natural and Environmental Science, Newcastle University, Newcastle upon Tyne NE30 4PZ, UK.; ^2^ School of Biology, University of Leeds, Leeds LS2 9JT, UK.; ^3^ Department of Biology and Marine Program, Boston University, Boston, MA 02215, USA.

## Abstract

Vertebrate growth is generally considered to be unidirectional, but challenging environmental conditions, such as heatwaves, may disrupt normal growth patterns and affect individual survival. Here, we investigate the growth of individual clown anemonefish, *Amphiprion percula*, during a marine heatwave. We measured the length of 134 wild clown anemonefish every month and monitored temperature at the scale of their anemone for five lunar months. Our results show that clown anemonefish shrink in response to heat stress and individuals that shrink also display relatively more catch-up growth. Further, shrinking is modulated by social rank and size, and individuals that shrink more often and in a coordinated fashion with their breeding partner have higher survival during the heat stress event. In conclusion, a plastic individual growth response to heat stress, constrained by the social environment, can lead to short-term survival benefits. If this plasticity were widespread in fishes, it may have marked consequences for populations and communities as heatwaves become more frequent.

## Teaser

Clown anemonefish survive heatwaves by shrinking together with their breeding partner.

## INTRODUCTION

Recent studies have revealed that environmental stressors can have marked effects on the growth of vertebrates, during both juvenile and adult stages (*1*–*5*). Environmental stressors, such as increasing temperature, have been linked to growth rate decline, increased size variability, ultimate size reduction, and even reversible shrinking (*6*–*11*). For example, adult marine iguanas (*Amblyrhynchus cristatus*) shrink in response to increased water temperatures, while young salmonids (*Salmo salar*) shrink at the start of winter (*12*, *13*). Bidirectional body size changes (growth and shrinking) allow site-attached individuals to match their metabolic demands to external conditions, such as when food availability decreases following environmental disturbance (*14*).

Social factors also influence growth, particularly among group-living vertebrates, where conflict among individual group members over social status and access to resources can drive size modification (*15*–*20*). For example, female meerkats (*Suricata suricatta*) have rapid growth spurts when they obtain dominant positions (*21*). Meanwhile, male cichlids (*Haplochromis burtoni*) experience stunted growth or shrinking when their social status is disrupted (*22*). This work has transformed our understanding of adaptive plasticity of vertebrate growth (*16*), but to date, no studies have investigated how both environmental and social conditions might interact to influence vertebrate growth.

The clown anemonefish, *Amphiprion percula,* and their magnificent sea anemone hosts, *Radianthus magnifica,* present an opportunity to investigate how challenging environmental and social conditions might interact to influence growth. Anemonefish live on Indo-Pacific coral reefs, where heat stress events are increasingly common and severe (*23*–*25*), while reef inhabitants often live close to their thermal tolerance limits (*26*, *27*). These heat stress events can have negative effects on anemones and their resident anemonefish (*28*, *29*). Clown anemonefish live in social groups, composed of a breeding pair with a dominant female (rank 1) and subdominant male (rank 2), and a small number of subordinate nonbreeders (ranks 3 to 6). The growth and size of clown anemonefish is related to environmental and social conditions: Dominant fish grow to match the size of their anemone and, presumably, the resources available to them (*15*, *30*, *31*); subordinate fish grow to maintain a specific size ratio with respect to their immediate dominant, thereby avoiding conflict and eviction, which bears a high likelihood of mortality (*32*–*35*).

Here, we investigate the growth and survival of clown anemonefish during a heat stress event. We studied 67 breeding pairs of a wild population of clown anemonefish in Kimbe Bay, Papua New Guinea, from February to August 2023. This spanned the world’s fourth global coral bleaching event, which caused temperatures in our study area to exceed the long-term average by 4°C (*36*). Body size change of individual clown anemonefish was measured once per lunar month, and temperature at the scale of individual anemones was measured every 4 to 6 days as an indicator of heat stress. First, we assessed (i) growth of clown anemonefish during the heat stress event, which revealed numerous unexpected observations of individuals shrinking. Subsequently, we investigated (ii) whether growth and shrinking during heat stress are related to initial size and social rank, (iii) how growth and shrinking relate to each other; (iv) whether growth and shrinking are predicted by heat stress; (v) whether shrinking is also predicted by social conflict; and (vi) whether patterns of shrinking and coordination within breeding pairs affect survival.

## RESULTS

### Clown anemonefish shrinking is common during a heat stress event

**Fig. 1.**
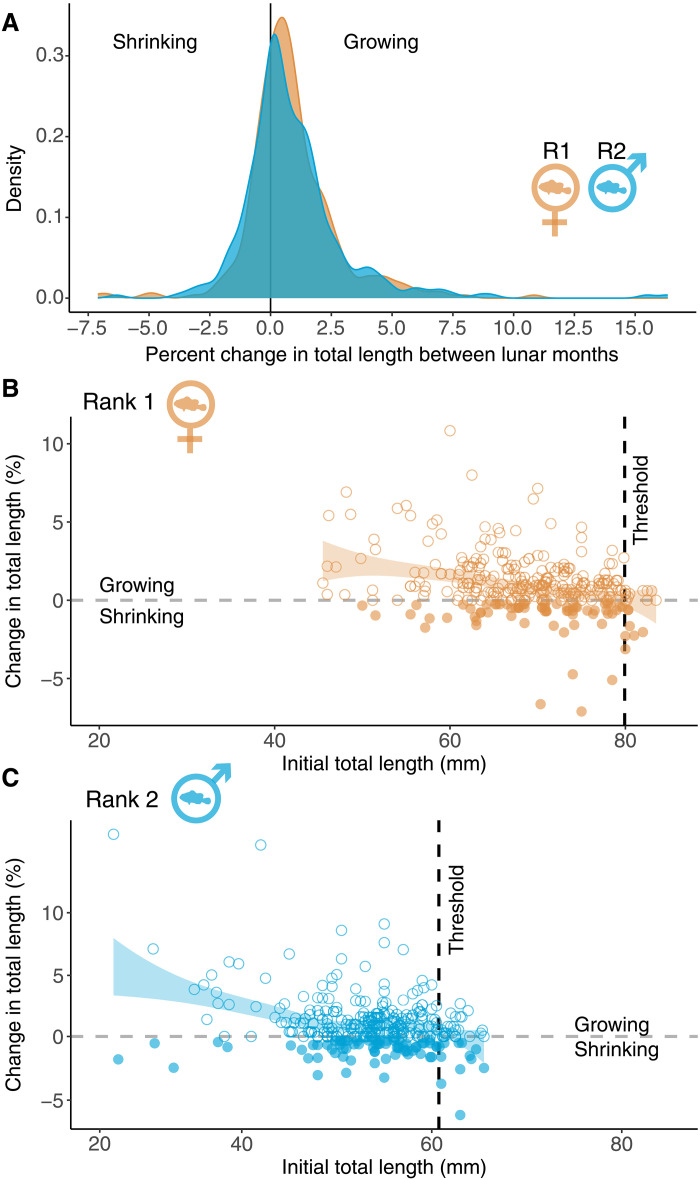
Change in total length (TL) of clown anemonefish during a heat stress event. The distribution of percent change in TL using repeated measurements across five lunar months (**A**), for rank 1 (female) clown anemonefish (orange), and for rank 2 (male) clown anemonefish (blue). Shrinking or negative growth responses are to the left of the 0 percent change mark (indicated by the black vertical line) and positive growth responses are to the right. (**B** and **C**) The modeled trends of percent change in TL in relation to initial TL using repeated measurements across five lunar months, for rank 1 (female) clown anemonefish (orange) (B) and for rank 2 (male) clown anemonefish (blue) (C). Positive growth is symbolized with open circles and shrinking is indicated with filled circles. The dashed gray line on the *y* axis indicates no change in percent change in TL. Regression tree analysis of percent change in TL as a function of initial TL was used to examine, per rank, at which initial TL threshold negative growth responses or shrinking is predicted (displayed as dashed black line on the *x* axis). The ribbons are loess smooths, showing general data trends.

We set out to examine growth of individual clown anemonefish during the 2023 heat stress event (*36*) in Kimbe Bay. To do this, we measured the total length (TL) of 134 individuals in 67 breeding pairs six times over five lunar months. We then plotted percent change in TL for rank 1 (female) and rank 2 (male) separately (*n*_rank 1_ = 322 observations; *n*_rank 2_ = 318 observations; see Materials and Methods). Notably, we found that clown anemonefish can shrink (*n*_shrinking_ = 151 observations) (Fig. 1, A to C). Of the 134 clown anemonefish, 71% of rank 1 individuals and 79% of rank 2 individuals shrank at least once (*n*_rank 1_ = 48; *n*_rank 2_ = 53). Of the shrinking fish, 59% shrank in only one lunar month (*n*_rank 1_ = 28; *n*_rank 2_ = 32), while 41% shrank multiple times (*n*_rank 1_ = 20; *n*_rank 2_ = 21) throughout the heat stress event. This suggests that shrinking in clown anemonefish is a common occurrence during marine heatwaves.

### Clown anemonefish growth and shrinking are related to size and social rank

Because growth patterns in clown anemonefish typically depend on initial size and social rank (*37*), we then investigated size change patterns among rank 1 and rank 2 individuals of different initial sizes. Shrinking of rank 1 occurred across almost the entire range of TLs (range_all rank 1_ = 50.00 to 83.50 mm TL, range_shrinking rank 1_ = 50.00 to 82.00 mm TL), while rank 2 shrinking occurred at all lengths (range_all rank 2 & shrinking rank 2_ = 27.00 to 65.5 mm TL). To examine the rank-dependent thresholds for shrinking, we used tree regressions. On average, rank 1 shrank when their initial TLs surpassed ~79.92 mm (predicted mean percent change in TL = −0.46, SE = 0.28; Fig. 1B), while rank 2 shrank at smaller sizes—initial TLs exceeding ~60.75 mm (predicted mean percent change in TL = −0.16, SE = 0.25; Fig. 1C). This result suggests that clown anemonefish growth and shrinking during a heat stress event depend on initial size and social rank of the individual.

### Clown anemonefish growth and shrinking are linked

**Fig. 2.**
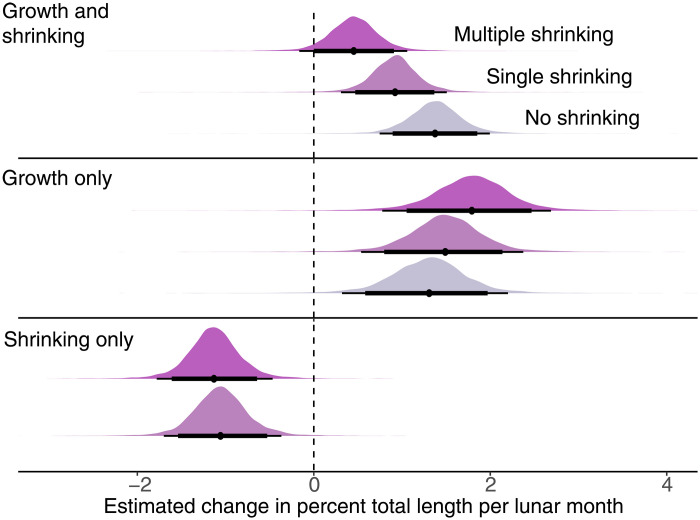
Percent change in TL for clown anemonefish with different shrinking response patterns. Bayesian models of the repeated measures of percent change in TL of clown anemonefish were run to estimate distributions of percent change in TL while considering individual response patterns. Groups are multiple shrinking individuals (dark purple), single shrinking individuals (light purple), or individuals displaying positive growth only (mauve), based on the observed frequency of shrinking. The estimated percent change in TL was modeled using this grouping predictor for all growth responses (*n* = 640) (top row), positive growth responses only (*n* = 489) (middle row), and shrinking responses only (*n* = 151) (bottom row). Predicted growth response estimates are displayed using density ridges of percent change in TL with 89 and 95% CIs. The dashed black line marks no change in percent TL on the *x* axis (*x* intercept = 0).

Next, we investigated how growth and shrinking are linked. There were three distinct response patterns in our population; some individuals did not shrink (25.20% of all fish), some shrank in only one lunar month (44.40%), and others shrank multiple times (30.40%). There were no apparent rank or sex differences in the frequency of these three response patterns (Fig. 1). To test whether growth patterns affected the total change in body length over the study period, we applied a Bayesian Generalized Linear Model framework. We found that, as expected, the clown anemonefish that shrank multiple times grew less over the entire study period than those that shrank only once or not at all (Fig. 2, tables S1 and S2, and model 1a). However, when we looked at growth in isolation, excluding occasions of shrinking, we found that multiple shrinkers also grew more compared to the other groups (median estimate, 95% high probability density (HPD) interval: multiple shrinkers = 1.79, 0.81 to 2.69; single shrinkers = 1.50, 0.56 to 2.33; no shrinkers = 1.31, 0.37 to 2.20; Fig. 2, tables S1 and S2, and model 1b). This result suggests that some individuals have an extraordinary level of growth plasticity, being both able to shrink and engage in catch-up growth.

### Clown anemonefish growth is predicted by heat stress

**Fig. 3.**
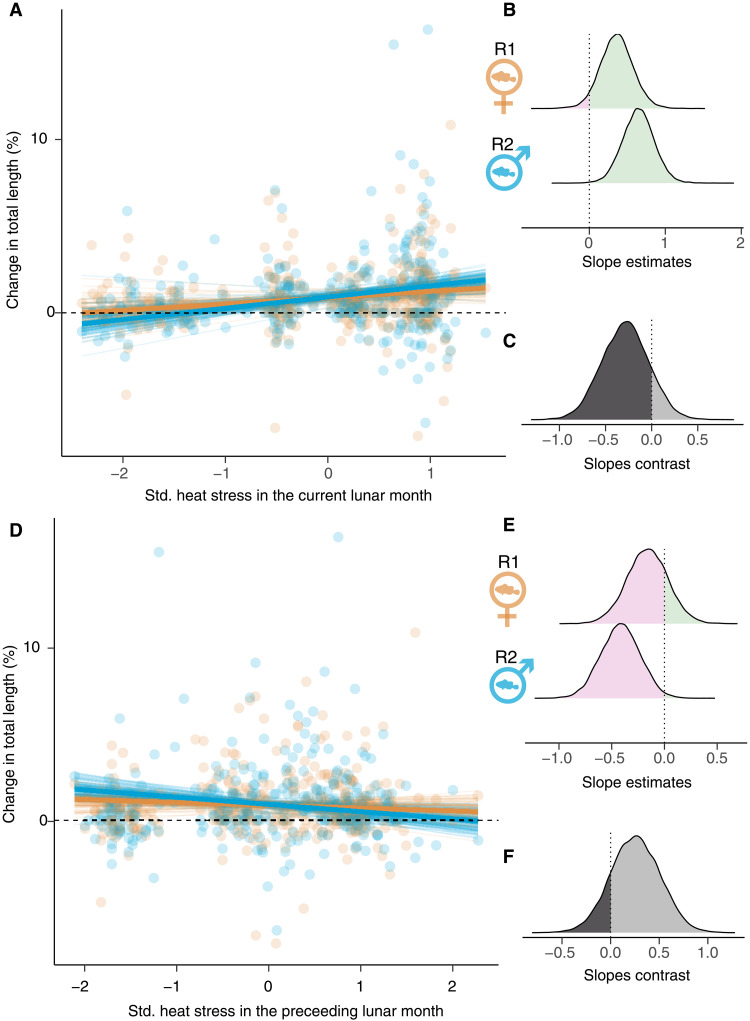
Change in body length of clown anemonefish in relation to heat stress. The percentage change in TL was fitted against (**A**) standardized (std.) temperature stress (mean = 0, SD = 1) in the lunar month of length measurements for rank 1 (in orange) and rank 2 (in blue), as well as against (**D**) temperature stress in the preceding lunar month. (A) and (D) show modeled change in TL as a function of heat stress, when fish TL and the other temperature stress measure are held at mean values. Each fine line is a modeled relationship from 1 of 100 model iterations and the bold lines represent the mean of these estimates. (**B** and **E**) show the distribution of the corresponding slope estimates for each rank, where pink indicates the portion of model estimates predicting negative relationships and green, positive relationships. (**C** and **F**) show the corresponding distribution of slope contrasts between ranks.

We next tested the hypothesis that individual growth is a function of heat stress, by comparing candidate Bayesian models of heat stress predictors measured at each anemone host (tables S1 and S2, models 2 to 12, and also see Materials and Methods). Our best model investigated the effect of heat stress (maximum temperature) at the scale of the anemone, in the current month and the preceding month, on percent change in TL. This analysis revealed a positive relationship between individual growth and individual heat stress in the current lunar month [change in TL in relation to maximum temperature in the same month: mean percent size change = 0.50, 95% credibility interval (CI) = 0.14 to 0.83] (tables S1 and S2 and model 7). However, the analysis also revealed a negative relationship between individual growth and heat stress at the anemone scale in the preceding lunar month (change in TL in relation to maximum temperature in the preceding month: mean percent size change = −0.29, 95% CI = −0.58 to −0.00) (tables S1 and S2 and model 7). We extended our model (model 13) to assess rank-specific responses on the predicted change in TL of clown anemonefish, which demonstrated a more pronounced growth response for rank 2 compared to rank 1, for both current and preceding maximum temperatures (Fig. 3, A to F, tables S1 and S2, and model 13). These results show that the growth of individual clown anemonefish is related to the heat stress that the individual experiences in both the current and preceding month, and that the social rank of the fish influences their responsiveness to heat stress.

### Clown anemonefish shrinking is also predicted by social conflict

**Fig. 4.**
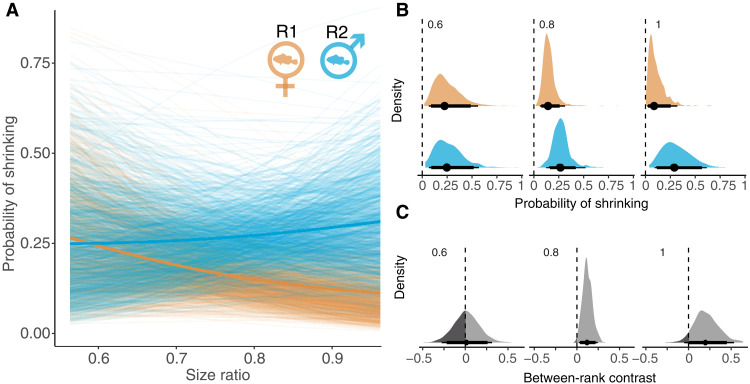
Shrinking likelihood in relation to size ratio of both clown anemonefish within a breeding pair. (**A**) The shrinking probability by the end of the lunar month was fitted against size ratios of the rank 2 to rank 1 at the start of that lunar month. Each fine line is a modeled relationship from 1 of 1000 model iteration and the bold lines represent the median of these estimates, for rank 1 (in orange) and rank 2 (in blue). (**B**) The distribution of model estimates for shrinking probability among rank 1 (in orange) and rank 2 (in blue) individuals, at three discrete size ratios—0.6 on the left, 0.8 in the middle, and 1.0 on the right. (**C**) The corresponding contrasts in shrinking probabilities between the two ranks show statistically significant differences in shrinking probabilities at larger size ratios.

In clown anemonefish experiencing normal conditions, dominants and subordinates regulate their growth to maintain well-defined size ratios between ranks to avoid costly conflict (*38*). This conflict would be exacerbated by shrinking that results in a reduction of pair size ratios, and we predicted shrinking could be a response to these interactions. To test this hypothesis, we fitted Bernoulli models with shrinking occurrence as the binary response (0 or 1), and pair size ratio, temperature in the preceding month, rank, and initial size as the predictors. Our results provide evidence for interacting effects of size ratio and rank on shrinking of clown anemonefish, beyond the effects of temperature and initial size, with rank 1 less likely to shrink when the pair is closer in size compared to when the pair is very different in size [rank 1 = 0.87, 0.64 to 1.17, 82.70%; rank 2 = 1.13, 0.75 to 1.69, 72.20% (median shrinking likelihood, 95% CI, direction of parameter estimate); Fig. 4, A to C, table S3, and model 14]. This finding suggests that the avoidance of social conflict also plays a role in determining whether clown anemonefish will shrink throughout a given lunar month.

### Clown anemonefish that shrink more often have higher chances of survival

**Fig. 5.**
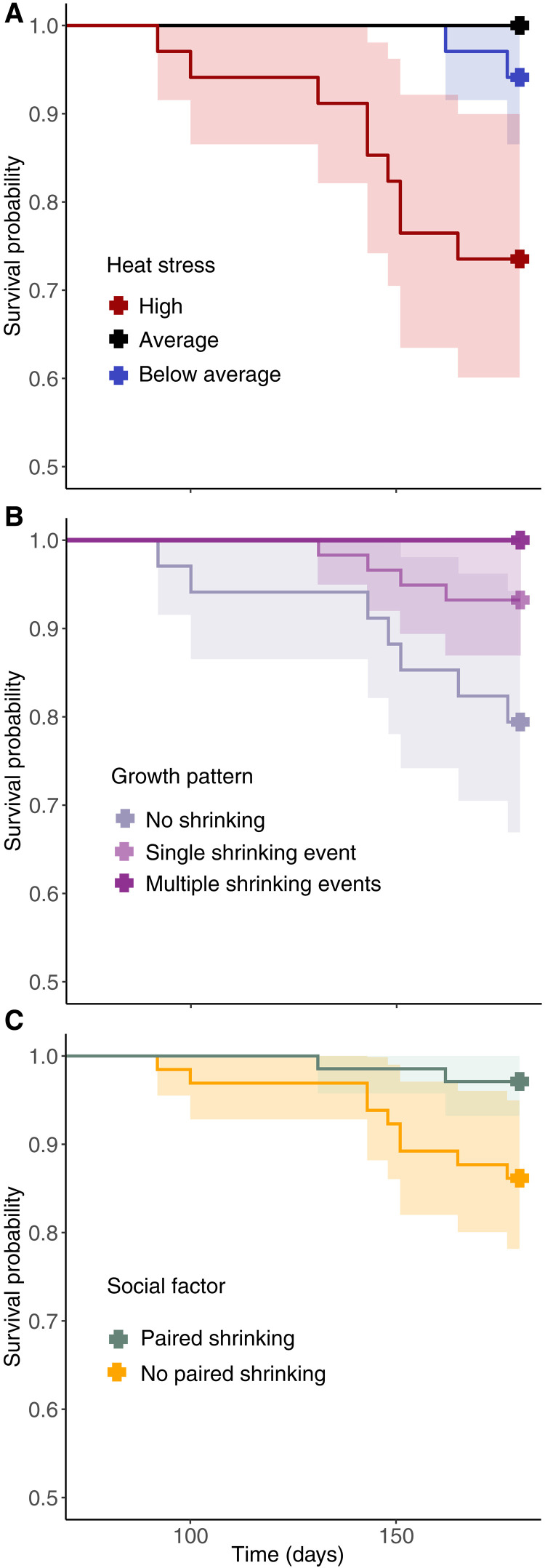
Survival of clown anemonefish. (**A** to **C**) Kaplan-Meier survival curves for heat stress at the anemone scale (A); growth patterns based on shrinking frequency (B); and social factors of shrinking within a breeding pair (C). (A) Levels of heat stress are represented by upper, mid, and lower quartiles of baseline water temperature around each host anemone over the study period (red is high heat stress, black is average heat stress, and blue is below average heat stress). (B) Survival for fish that did not shrink (mauve), shrank once (light purple), or shrank multiple times (dark purple). (C) Survival probabilities when shrinking as a breeding pair (i.e., social response of both fish shrinking—green) versus only one individual or no individuals within the breeding pair shrinking (yellow). Survival is calculated in daily time steps, up until the event (i.e., mortality or survival), and ribbons show 95% confidence intervals around estimated survival probabilities. Survival probabilities at the end of the study are indicated with bold, color-filled plus symbols.

Eleven individuals in the population died during the heat stress event (*n*_rank 1_ = 5, *n*_rank 2_ = 6). We tested the hypothesis that heat stress measured at the scale of the anemone negatively affects the likelihood of survival of individual fish in that anemone, using a survival analysis. This analysis revealed that higher minimum temperatures (reflecting heat persistence over the entire study period) decreased clown anemonefish survival probability [χ^2^(1) = 44.13, *P* < 0.001, β = 1.60, SE = 0.28; Fig. 5A]. Subsequently, we tested the hypothesis that singular and multiple shrinking will mitigate the effect of heat stress, using survival analysis (table S4 and models 15 to 19). Individuals that shrank within the study period were more likely to survive (Fig. 5B); a single shrinking event increased an individual’s survival probability by 78% compared to individuals that did not shrink, and all clown anemonefish that shrank multiple times survived to the end of the study period [χ^2^(1) = 10.89, *P* = 0.001, β = −1.52, SE = 0.53, hazard ratio (HR) = 0.22]. Last, because shrinking that causes a reduction in pair size ratios should lead to social conflict, which is an additional cause of mortality, we tested the hypothesis that coordinated shrinking will mitigate this effect. The initial size or rank of an individual did not affect their survival, but when both partners within a breeding pair shrank (i.e., paired shrinking), survival probabilities were higher than when only one or neither of the individuals within their pair shrank [χ^2^(1) = 5.64, *P* = 0 0.020, β = −1.62, SE = 0.78; Fig. 5C]. Collectively, these results indicate that individual shrinking and shrinking in a coordinated fashion during a heat stress event enhance survival.

## DISCUSSION

Vertebrate shrinking has been documented in just a few taxa, and our understanding of how a reduction in body size during adult stages is related to environmental and social factors is limited (*8*, *11*–*13*, *21*, *22*). We find that clown anemonefish can adjust their size rapidly in response to their thermal environment. Growth plasticity varies among individuals, with a better ability to respond to local environmental conditions resulting in higher survival. Our results show that shrinking is a strategy that adult marine fish can use to increase survival in the face of environmental disturbance, in line with previous work demonstrating the dynamic nature of size change in juvenile fish (*13*). However, our study also emphasizes that even as they respond to heat stress, the social environment of the fish impacts their growth response. The breeders’ probability of shrinking is related to the size ratio between the female and the male. This is in line with known social constraints on growth that maintains well-defined size hierarchies in clown anemonefish (*15*, *16*, *33*, *35*), and additionally highlights the complex interaction between ecological and social drivers of growth plasticity in social vertebrates.

High growth plasticity may be favored by natural selection in situations where environmental variation cannot be escaped. The growth and shrinking responses found in our population appear complex, with individuals that shrink also engaging in catch-up growth. Plasticity of growth and size in response to variation in temperature will be especially useful for organisms that are very site attached and cannot regulate heat stress behaviorally and that live close to their thermal maximum in fluctuating environments (*39*–*41*). This is the case for clown anemonefish, which are obligately associated with anemone hosts in tropical shallow water ecosystems (*34*). Given our results, we hypothesize that individual shrinking in response to heat stress will be found in other site-attached, small bodied, tropical species. Testing this idea will require monitoring of the body size of marked individuals and the temperatures that they experience at appropriate temporal and spatial scales.

Our study includes no information on the mechanism of shrinking but previous studies on vertebrate growth may provide some insight on the physiological costs of being large as temperatures increase. Reducing body length through tissue resorption, as opposed to simply losing weight, may be adaptive because it allows for the adjustment of metabolic demand and maintenance of condition and performance (*14*, *42*, *43*). Shrinking may mediate the mismatch of increased metabolic rates and lower oxygen availability at higher temperatures: The gill oxygen limitation theory suggests that oxygen uptake in fishes is limited by the two-dimensional surface of the gills, which cannot keep pace with increased three-dimensional body size, particularly at higher temperatures (*44*). Alternatively, the effect of heat stress may be indirect, mediated via changes in food availability or composition. If heat stress events reduce food supplies or require increased feeding effort, it may pay to be smaller because larger individuals are more likely to have high metabolic demands (*45*–*47*). In either case, we can only speculate at this stage as to how the fish shrink to meet their changed physiological demands induced here by a combination of heat stress and social position. It is possible that neuroendocrine pathways are triggered, for example, via thyroid hormones, which are negatively correlated with heat stress in fish and are known to regulate growth (*48*, *49*). Testing these hypotheses will require rigorous laboratory experiments cross-factoring heat stress and food availability, in conjunction with appropriate physiological measurements.

The individual shrinking described here will have population-level consequences. The upside of shrinking for the population is that it reduces the death rate during the heat stress event, which may facilitate population persistence during marine heatwaves that are increasing in frequency and intensity in tropical oceans (*23*, *25*). The downside of shrinking for the population is that it will decrease the birth rate as the fish become smaller, because reproductive output is related to body size in anemonefishes and most other fishes (*50*–*52*). Another potential downside of smaller fish is that they might be poorer mutualistic partners, if they provide their anemone with less protection or less nutrients, though, to date, there is only evidence that the number and not the biomass of fish matters for this mutualistic interaction (*53*).

More generally, reductions in body size of communities, populations, and individuals are widely recognized as an ecological response to climate change in terrestrial and aquatic systems (*54*–*58*). Reduced body size has been called the third universal ecological response to global warming, along with shifts in species’ ranges and changes in phenology (*54*, *59*–*62*). At the community level, reductions in body size can be explained by shifts in species composition (Bergmann’s rule) and/or shifts in population body size (James’ rule); At the population level, reductions in body size can be explained by individuals growing faster as juveniles but having smaller sizes as adults under warming, such that each cohort is smaller than the one before it as Earth warms [temperature size rule (*54*, *57*, *63*)]. The latter can be viewed as an evolutionary or plastic response to warming (*54*, *55*), but it is rarely considered that the current cohort of adults can themselves shrink. This is the extraordinary growth plasticity in response to heat stress that we demonstrate here: Adult individuals shrink. If such individual-level shrinking were widespread, it would provide a plausible alternative hypothesis for the rapidly declining size of fish in the world’s oceans (*43*, *54*, *64*, *65*).

## MATERIALS AND METHODS

### Experimental design

The study was carried out on a wild population of clown anemonefish in Kimbe Bay, Papua New Guinea (5.16°S, 150.50°E). Clown anemonefish have been extensively studied, including research into their life stages, life expectancy, the stability of associations between the resident fishes and their hosting anemone, and their complex social structures (*16*, *66*–*68*). This thorough understanding makes them an attractive model system with which to study how both social and environmental factors affect individual-level growth plasticity. We focused on breeding pairs, meaning the largest and dominant rank 1, which is a female, and the second largest male (rank 2) within a social group, as their rank can be easily distinguished due to their strict size hierarchy (*15*), and because lower ranking individuals are more at risk of eviction (*32*). We selected only those breeding pairs living on single *R. magnifica* anemones [formerly *Heteractis magnifica* (*69*)] to reduce variation in microhabitat. Sixty-seven groups of clown anemonefish were observed every 4 to 6 days for 180 days across five lunar months between February and August 2023, where all groups were given unique tags and individual ranks on a host could be differentiated through a combination of color markings and relative body size. Ethics for all research included in this study was granted by the Animal Welfare Ethical Review Body (AWERB) at Newcastle University (AWERB ID: 998).

At the start of the study, all individual clown anemonefish were caught underwater using hand nets, photographed for unique markings (*32*), and measured for TL to the nearest 0.5 mm using calipers (*31*), before returning individuals to their host anemone. At the start of the study, mean (±SE) TL was 67.50 ± 1.10 mm for rank 1 (females) and 52.60 ± 0.91 mm for rank 2 (males). At the end of the study period, the dataset included 640 observations of the TLs of 134 individuals.

To measure the temperature experienced by individual groups, a HOBO MX2202 temperature logger, programmed to measure at 2-min intervals, was placed next to the anemone host at every visit to the breeding pair (every 4 to 6 days for 180 days). We averaged the temperature readings taken at each anemone microhabitat on a given survey day to control for time spent at the anemone (mean ± SD = 7 ± 4 min per survey day). To increase statistical power, current temperatures for lunar month 5, and the preceding temperatures for the first lunar month, were projected from available temperature data using autoregressive integrated moving average (ARIMA) time series analysis (package “forecast”). We calculated and compared three measures to proxy the heat stress experienced by the breeding pairs on each host anemone per lunar month: maximum temperature, mean temperature, and the range in temperature (table S5).

To calculate the change in TL of clown anemonefish, all individuals were caught and measured six times; once before the start of the study, and subsequently at the end of each of the five lunar months. Anemonefish were caught using hand nets and measured using calipers underwater. TL was selected over alternate body size estimates, as TL can be most consistently measured across observers and life stages (*70*), thus optimizing accuracy. In addition, measurements were repeated three times per individual. If any one measurement was more than 1 mm different to the other two, all measurements were repeated for the individual to minimize measurement error. Measurements were then averaged to obtain the TL for the end of a given lunar month, which was subtracted from the TL at the start of that lunar month to calculate absolute difference in TL. This value was divided by the initial TL of the anemonefish (i.e., the TL at the start of that lunar month) and multiplied by 100 to get the percentage change in TL, where a negative value indicates shrinking. To summarize growth patterns, we developed three measures:

1) Shrinking within a breeding pair: A binary variable to indicate whether any observed shrinking was present in breeding pairs. A value of 1 indicates that shrinking was observed for both individuals within a breeding pair throughout the study, regardless of whether shrinking occurred at the same time or with a time lag for each member of the breeding pair. No further distinctions were made into time-lagged responses of shrinking within the breeding pair, as the study cannot capture all shrinking before the study start or shrinking after the study period of 180 days.

2) Size ratios of rank 1 and rank 2 within their breeding pair: The ratio of the TL of rank 2 to its dominant rank 1 at the start of a given lunar month was used to assess how close in size two anemonefish within their breeding pair were. Size ratios are known to be strictly upheld in clown anemonefish, and noncompliance with size ratios can be met with aggression and potential eviction from the anemone host (*33*, *35*). The size ratios among wild populations are thought to generally be 0.8 (*33*), while lab-reared breeding pairs can be much closer in size (*35*).

3) Growth patterns: A categorization of individual growth patterns into the following: 1 = no shrinking events to indicate a “no-shrinker,” 2 = a single shrinking event to indicate a “single-shrinker,” or 3 = multiple shrinking events throughout the study period, referred to as a “multiple shrinker.” To ensure statistical power, no further distinctions were made to differentiate between consecutive or nonconsecutive shrinking events, nor did we further distinguish the number of shrinking events across individuals that demonstrated multiple shrinking events.

Mortality of clown anemonefish was assumed when an individual was not seen on its home anemone for three consecutive survey rounds. While we did not observe mortality directly, we are confident that these occasions of disappearance represent mortality for several reasons: (i) Clown anemonefish are extremely site attached and face harsh ecological constraints that prevent them from switching anemones (*34*, *38*, *71*, *72*); (ii) all suitable anemones in our study areas are occupied, leaving no space to emigrate to; and (iii) we thoroughly surveyed all anemones in a given area and took pictures for identification every lunar month. At the end of each lunar month, all individuals were caught and processed as described above. If a fish had been recorded as missing, the end of the lunar month would be used to confirm mortality of that individual. The time between initial date of marked absence and the last recorded date at which the individual was still present within the breeding pair would be transformed into a time of event range within which the mortality event occurred.

### Statistical analysis

All statistical analysis was carried out using R version 4.4.1 (2024-06-14 ucrt) (*73*). To explore size and rank in relation to shrinking, regression trees (package: “tree”) were run independently for rank 1 and rank 2 clown anemonefish. The percent change in TL of individuals between lunar month 1 and 5 (*n*_rank 1_ = 322, *n*_rank 2_ = 318) was modeled against initial TL, to examine thresholds and predicted shrinking for each of the ranks.

We used Bayesian generalized linear models to investigate the associations of shrinking to heat stress, social factors, and growth patterns [packages: “tidyverse,” “brms,” “tidybayes,” “modelr,” “coda,” and “cmdstanr” (*74*)]. A Bayesian modeling approach allowed us to explore the relationships within complex models using a relatively small dataset (*75*) and accounts for uncertainty of measurement methods (*76*). For these models, percent change in TL was the response, and predictor variables were systematically introduced to build and compare candidate models. All noncategorical predictor variables were centered and scaled to 1 SD (mean = 0, SD = 1), and all models included grouping effects to control for the repeated measures of individual anemonefish as well as the lunar months. Weakly informative and ecologically plausible priors were used, following prior predictive checks (table S9). All models were screened for model convergence, adequate effective sample sizes (ESS bulk and tail values > 1000), suitable model shrinking (R-hat values <1.05), and autocorrelation values [auto correlation function (ACF) plots], and were validated using leave-one-out cross-validation (pareto *k* values <0.7) (packages: “posterior” and “bayesplot”). The performance of candidate models was compared using leave-one-out information criterion (LOO-IC) scores and pseudo *R*^2^ values (packages: “loo” and “bmrs”). To confirm effects, candidate predictor models were compared with LOO-IC to null and reduced models (models 11 and 12; see tables S1 and S5), with the null model including only the grouping effects, while the reduced model included the grouping effects and the additional covariates initial TL and rank, and their interaction. To assess support for effect directions and effect differences among predictors, pairwise and grouped effect comparison and contrast tools were used (packages: “emmeans,” “ggeffects,” “bayestestR,” and “parameters”; see table S2).

To inspect the relationship between change in TL and heat stress, we developed models that compared change in TL with measures of heat stress exposure. We compared models that included (i) preceding heat stress exposure (i.e., the temperature measured during a preceding lunar month compared to the TL change of clown anemonefish during a given lunar month), (ii) current heat stress exposure (i.e., the temperature measures during a given lunar month compared to the TL change of clown anemonefish during that same lunar month), and (iii) both preceding and current heat stress exposure (models 2 to 10; see tables S1 and S2). As models performed similarly for each of the candidate heat stress measures (see table S5), we selected maximum temperature as a reliable and key indicator of heat stress (*25*, *77*, *78*) and looked at both preceding and current heat stress exposure to investigate time-lagged responses to heat stress (model 13; see tables S1 and S2).

To test whether the three observed growth patterns predict growth and shrinking of individual clown anemonefish, growth pattern was used as a predictor variable (model 1a to c; see tables S1 and S2) for all size change observations, for positive growth observations only, and for shrinking observations only, to discern differences in growth rates between no shrinkers, single shrinkers, and multiple shrinkers.

To investigate whether size hierarchies remain stable among breeding pairs during heat stress exposure, or whether TL change in response to heat stress is driven largely by individual constraints, a Bernoulli model was fitted with shrinking by the end of a given lunar month as response and size ratio of the rank 2 to rank 1 in their breeding pair at the start of a given lunar month as predictor (model 14; see tables S2 and S3). We also included the other predictors previously described to control for their effects on the outcome. The analyses used the same packages as described for the other Bayesian models, as well as “parameters.”

Last, Cox hazard ratio models of survival were fitted to investigate how heat stress, initial TL, rank, shrinking within breeding pairs, and growth patterns affect the survival of clown anemonefish [models 15 to 19; see table S4, packages: “survival” and “survminer,” using time to event, and left-censored data (*79*)]. Clown anemonefish are long-lived and typically experience slow turnover in their populations (*50*). While mortality during our study was high among breeding pairs, with 11 mortality events, low relative occurrence overall restricted the complexity of models, meaning that sequential addition of predictor variables or their interactions would not be informative. Predictors required transformation to adhere to model assumptions of survival outcomes, and models were fitted separately. Model constraints required exclusion of one breeding pair (“B003”), as data transformations were not possible for rank 2. To assess heat stress over the entire study period, minimum water temperatures at the level of the host anemone was selected to best reflect thermal accumulation and heat persistence (*80*). High heat stress was represented by the 75th quantile of this minimum water temperature around the anemone, medium heat stress was the 50th quantile, and low heat stress was the 25th quantile. Similarly, to estimate the initial TL of the individual, TL at the start of the study was used to standardize this measure across all individuals, at the cost of interindividual variance throughout lunar months. Rank, shrinking within breeding pairs, and growth patterns remain stable across the study period and needed no transformations. All survival models were tested for predictive fit and model assumptions (see table S8).

### AI-assisted technologies

During the development of R scripts for data management, data analysis, and data visualization, OpenAI’s ChatGPT (version ChatGPT-4) assisted with coding and debugging. Prompts used when working with the tool included “check the current code for errors and issues given the following structure of the data frame: [with details of the developed code, data frame structure, and any error messages]. Give alternatives, explanations, and considerations,” “explain the following error message and how to resolve: [with details of the code used and the error message],” “explain more about the following package in R studio: [package details] and describe data structure requirements and common mistakes made,” and “provide an alternative way to calculate the following: [with details of desired outcome and code used].” The artificial intelligence (AI) tool highlighted and explained errors, provided corrections to code, and suggested alternative solutions.
