# Supplementary material for: Individual clown anemonefish shrink to survive heat stress and social conflict
Source: Sci Adv. 2025 May 21;11(21):eadt7079. doi: 10.1126/sciadv.adt7079 (PMC12094202; doi:10.1126/sciadv.adt7079)
Supplement: Supplementary file 1 — Legends for tables S1 and S5 Tables S2 to S4 and S6 to S9 [file sciadv.adt7079_sm.pdf]

Science Advances
AAAS

Supplementary Materials for

Individual clown anemonefish shrink to survive heat stress and social conflict

Melissa A. Versteeg *et al.*

Corresponding author: Melissa A. Versteeg, m.versteeg5@gmail.com;
Theresa Rueger, theresa.rueger@newcastle.ac.uk

Sci. Adv. **11**, eadt7079 (2025)
DOI: 10.1126/sciadv.adt7079

The PDF file includes:

Legends for tables S1 and S5
Tables S2 to S4 and S6 to S9

Other Supplementary Material for this manuscript includes the following:

Tables S1 and S5

Supplementary Materials

Extended details of Bayesian models and Cox Hazard survival models, including model fit and model assumption test results.

Table S1.

(see **Auxiliary files**)

*Note**: Model 1b (n = 489) and 1c (n = 151) include growth and shrinking observations only.

Model effect estimates investigating heat stress impacts, social status, and growth patterns on size change metrics. The table summarizes the posterior estimates for models assessing the effects of various temperature metrics (current, preceding, and their combinations), social rank, and growth patterns, on parameters of percent change in size. Each model includes both population-level and group-level (random) effects, including intercepts and coefficients for temperature (i.e., heat stress), rank, and initial total length (TL), as well as their interactions. The estimates are presented with standard errors (in brackets) and 95% credibility intervals (CIs). Sigma values represent the residual standard deviation of the models.

Table S2.

	Model 1a	Model 1b	Model 1c	Model 7	Model 13
emmeans (HPD)					
Rank 1				1.44<br>(1.04 - 1.83)	1.45<br>(1.05 - 1.83)
Rank 2				0.16<br>(-0.26 - 0.58)	0.17<br>(-0.25 - 0.59)
No shrinking	1.38<br>(0.74 - 2.02)	1.31<br>(0.37 - 2.20)			
Single shrinking	0.92<br>(0.31 - 1.50)	1.50<br>(0.56 - 2.33)	-1.06<br>(-1.71 - -0.37)		
Multiple shrinking	0.45<br>(-0.19 - 1.04)	1.79<br>(0.81 - 2.69)	-1.13<br>(-1.81 - -0.48)		
Trends (HPD) Temperature: current					
Rank 1				0.50<br>(0.14 - 0.83)	0.35<br>(-0.07 - 0.79)
Rank 2				0.50<br>(0.14 - 0.83)	0.64<br>(0.21 - 1.07)
Trends (HPD) Temperature: preceding					
Rank 1				-0.29<br>(-0.58 - -0.00)	-0.16<br>(-0.54 - 0.23)
Rank 2				-0.29<br>(-0.58 - -0.00)	-0.42<br>(-0.81 - -0.06)
Trends (HPD) Initial size					
Rank 1				-0.79<br>(-1.11 - -0.50)	-0.81<br>(-1.11 - 0.50)
Rank 2				-1.03<br>(-1.38 - -0.67)	-1.02<br>(-1.39 - -0.67)
Contrast (HPD)					
Rank 1 - Rank 2<br>(social status)				1.28<br>(0.82 - 1.76)	1.28<br>(0.82 - 1.75)
Rank 1 - Rank 2<br>(temperature: current)				0	-0.29<br>(-0.80 - 0.20)
Rank 1 - Rank 2<br>(temperature: preceding)				0	0.26<br>(-0.22 - 0.79)
Rank 1 - Rank 2<br>(initial size)				0.24<br>(-0.20 - 0.68)	0.21<br>(-0.22 - 0.65)
No shrinking - Single shrinking	0.46<br>(0.06 - 0.88)	-0.19<br>(-0.63 - 0.24)			
No shrinking - Multiple shrinking	0.92<br>(0.48 - 1.35)	-0.49<br>(-1.00 - -0.00)			
Single shrinking - Multiple shrinking	0.47<br>(0.10 - 0.86)	-0.30<br>(-0.74 - 0.16)	0.08<br>(-0.31 - 0.46)		

Effect sizes, contrasts, and trends across models including heat stress exposure, social factors, and growth patterns. This table presents the estimated marginal means (emmeans) and high posterior density (HPD) intervals for rank and growth conditions under varying models. The models assess the impact of heat stress (current and preceding heat stress), social rank, and initial size, as well as growth patterns of no shrinking, single shrinking, and multiple shrinking. Trends are reported separately for current and preceding heat stress, as well as initial size of clown anemonefish. Contrasts are provided to illustrate the differential effects of social status and shrinking on change in size under heat stress. The HPD intervals provide the 95% credibility intervals for each estimate.

Table S3.

	Model 14: Size ratio R2:R1		
	Median	95 % CI	PD %
Regression coefficient			
Intercept	0.20	0.09 - 0.54	99.54
Size ratio R2:R1 (scaled)	0.87	0.64 - 1.17	82.73
Temperature: preceding (scaled)	1.19	0.79 - 1.87	77.68
Rank	2.07	1.13 - 3.92	99.16
Initial TL (scaled)	1.42	1.03 - 1.98	98.50
Size ratio R2:R1 (scaled) x Rank	1.13	0.75 - 1.69	72.17

Model results assessing shrinking by the end of a lunar month and size ratios at the start of a lunar month within breeding pairs. The table displays the results from a Bernoulli model evaluating the effects of size ratios within breeding pairs of clown anemonefish, heat stress, and rank on shrinking responses within breeding pairs by the end of a lunar month. Regression coefficients are presented for the intercept, size ratio, preceding heat stress, rank, and initial total length (TL), along with their interactions. The estimates are reported with 95% credibility intervals (CIs) as well as the probability direction (PD, %). The model includes random effects for group ID and lunar month.

Table S4.

	Model 15: Heat stress	Model 16: Initial size	Model 17: Rank	Model 18: Shrinking of both individuals within a breeding pair	Model 19: Growth patterns
Model parameters					
Coefficient	1.60	-0.09	0.57	-1.62	-1.52
Exponential coefficient	4.97	0.92	1.78	0.20	0.22
Exponential -coefficient	0.20	1.09	0.56	5.04	4.57
Standard error	0.28	0.30	0.62	0.78	0.53
Z-value, p-value	5.73, < 0.001	-0.29, 0.78	0.92, 0.36	-2.07, 0.04	-2.84, 0.004
95 % CI	2.87 - 8.61	0.50 - 1.66	0.52 - 6.07	0.04 - 0.92	0.08 - 0.62

Cox Hazard models of survival of clown anemonefish. The table includes the results from Cox proportional hazards models assessing the impact of various factors on clown anemonefish survival. Models assess the effects of heat stress (Model 15), initial size (Model 16), rank (Model 17), shrinking of both individuals within a breeding pair (Model 18), and growth patterns (Model 19). Coefficients, exponential transformations of the coefficients (hazard ratios), standard errors, z-values, p-values, and 95% confidence intervals (CIs) are displayed.

Table S5.
(see **Auxiliary files**)

*Note***: Model 1b and 1c include growth, and shrinking observations only, and therefore cannot be compared for model fit.

Model fit and comparisons for models of heat stress, social factors, and growth patterns. This table provides an overview of the model fit statistics and comparisons across models examining the effects of heat stress, social factors, and growth patterns. The models are evaluated using Bayesian R^2, expected log pointwise predictive density (elpd_loo), effective number of parameters (p_loo), and the leave-one-out information criterion (looic), including standard errors. Expected log pointwise predictive density differences (elpd_diff) with standard errors are reported for model comparisons.

Table S6.

	Model 14: Size ratio R2:R1
Bayes R^2	0.05
elpd_loo (s. e.)	-330.90<br>(13.10)
p_loo (s. e.)	15.60<br>(1.00)
looic (s. e.)	661.80<br>(26.30)

Model fit for shrinking probabilities and size ratios within breeding pairs. The table includes the model fit statistics for the analysis of shrinking probabilities in relation to size ratios within breeding pairs, including Bayesian R^2, expected log pointwise predictive density (elpd_loo), effective number of parameters (p_loo), and the leave-one-out information criterion (looic).

Table S7.

	Model 14: Size ratio R2:R1	
	Predicted probabilities	95% CI
Size ratio rank 2 to rank 1 (scaled)		
Rank 1		
-6	0.29	0.05 - 0.76
-4	0.24	0.06 - 0.60
-2	0.19	0.07 - 0.43
0	0.15	0.07 - 0.33
2	0.12	0.04 - 0.30
4	0.09	0.02 - 0.32
Rank 2		
-6	0.22	0.03 - 0.71
-4	0.24	0.06 - 0.61
-2	0.25	0.09 - 0.52
0	0.26	0.13 - 0.49
2	0.28	0.12 - 0.52
4	0.30	0.09 - 0.63

Predicted probabilities of shrinking based on size ratios within breeding pairs. Predicted probabilities of shrinking for individual clown anemonefish within breeding pairs, based on the scaled size ratio between rank 2 and rank 1. Probabilities are presented for scaled size ratios, along with their corresponding 95% confidence intervals (CIs).

Table S8.

	Model 15: Heat stress	Model 16: Initial size	Model 17: Rank	Model 18: Shrinking of both individuals within a breeding pair	Model 19: Growth patterns
Value (standard error)					
Concordance	0.87 (0.07)	0.53 (0.09)	0.57 (0.07)	0.67 (0.06)	0.76 (0.05)
Value, df, p-value					
Likelihood ratio	44.13, 1, < 0.001	0.08, 1, 0.80	0.87, 1, 0.30	5.64, 1, 0.20	10.89, 1, 0.001
Wald test	32.84, 1, < 0.001	0.08, 1, 0.80	0.84, 1, 0.40	4.28, 1, 0.40	8.08, 1, 0.004
Logrank test	81.10, 1, < 0.001	0.08, 1, 0.80	0.86, 1, 0.40	5.29, 1, 0.20	10.24, 1, 0.001
X^2, df, p-value					
Model assumption test	0.01, 1, 0.93	0.60, 1, 0.44	0.24, 1, 0.62	0.00, 1, 0.96	0.01, 1, 0.92

Model fit and assumptions for Cox Hazard survival models. This table presents the Cox Hazard survival model fit statistics and assumption tests. Concordance values are presented to indicate correct ranking of survival times, likelihood ratio, Wald, and Logrank tests evaluate the significance of the predictors, and model assumption tests (X^2, df, p-value) indicate whether the proportional hazards assumption is met.

Table S9.

Models	Priors
1-14:	
- Intercept	Student’s t (3,0,3)
- Coefficient	Normal (0, 4)

Model priors. The table includes details of the priors used for the statistical models.
